# Addressing the Challenge: A Review of Effective Prevention Strategies for Childhood Obesity in India

**DOI:** 10.7759/cureus.56257

**Published:** 2024-03-16

**Authors:** Pankaj C Jambholkar, Abhishek Joshi, Sonali G Choudhari

**Affiliations:** 1 Community Medicine, Jawaharlal Nehru Medical College, Datta Meghe Institute of Higher Education and Research, Wardha, IND

**Keywords:** stakeholders, interventions, public health, india, prevention strategies, childhood obesity

## Abstract

Childhood obesity is a growing public health concern in India, with rising prevalence rates and associated health risks. This review examines effective prevention strategies for addressing this issue. Through a comprehensive analysis of research findings, policy initiatives, and community-based interventions, the review identifies critical components of successful prevention efforts. These include multi-sectoral collaborations, tailored interventions addressing socioeconomic and cultural factors, and the involvement of families and healthcare professionals. The importance of addressing childhood obesity in India is underscored, given its significant impact on health outcomes, healthcare costs, and quality of life. The review concludes with a call to action for stakeholders and policymakers to prioritise prevention efforts, allocate resources, and implement evidence-based interventions to combat childhood obesity effectively. By working together, India can mitigate the adverse effects of childhood obesity and promote a healthier future for its children.

## Introduction and background

Childhood obesity has reached epidemic levels in developed as well as in developing countries. According to recent studies, the prevalence of childhood obesity in India has risen alarmingly, with both urban and rural areas affected [1]. The prevalence of obesity was 19.7% and affected about 14.7 million children and adolescents [1]. This trend is concerning due to its association with various health complications and its long-term impact on the well-being of individuals [2]. Implementing effective prevention strategies to combat childhood obesity cannot be overstated. Obesity during childhood not only increases the risk of developing chronic diseases such as diabetes, cardiovascular diseases, and hypertension but also has psychological and social implications. Preventive measures are crucial not only for the health and well-being of children but also for reducing the burden on healthcare systems and promoting a healthier future generation [3].

This review explores and evaluates effective prevention strategies for childhood obesity in India. By examining current research, policy interventions, and community-based initiatives, the review seeks to identify successful approaches and highlight areas for improvement. Ultimately, the goal is to provide insights and recommendations to inform the development and implementation of comprehensive obesity prevention programs tailored to the Indian context.

## Review

Prevalence and causes of childhood obesity in India

Statistics on Childhood Obesity Rates

The escalating prevalence of childhood obesity in India has become a significant concern. A meta-analysis spanning 21 studies conducted from 2003 to 2023, encompassing 186,901 children, revealed a pooled prevalence of childhood obesity at 8.4%, albeit with notable heterogeneity. This prevalence exhibits considerable variability across different regions and demographics within India, fluctuating between 3% and 22.8% among school children aged 6 to 16 years [1]. Moreover, the incidence of overweight children under the age of five has been on the rise, surging from 2.1% (during 2015-2016) to 3.4% (throughout 2019-2021) [4]. Numerous studies have posited potential causes for this upward trend in overweight and obesity among Indian children, with attributing factors such as inadequate physical activity, prolonged television viewing, urban residence, and socioeconomic status within families [4]. The aetiology of childhood obesity in India is multifaceted, stemming from genetic predispositions, behavioral patterns, environmental influences, and socioeconomic circumstances [1]. Given the burgeoning burden of childhood obesity, there is a need for a sustained, multi-sectoral response involving public, private, and healthcare sectors. Various prevention strategies have been proposed to address this challenge, including promoting increased physical activity, curbing sedentary behaviors, implementing personalized nutrition plans, and launching community-based interventions [5].

Socioeconomic Factors Contributing to Obesity

**Figure 1:**
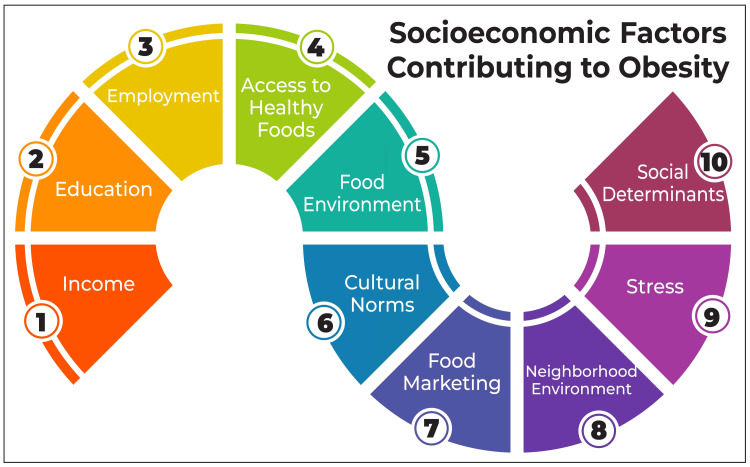
Socioeconomic factors

Socioeconomic factors play a pivotal role in driving the prevalence of childhood obesity in India. While children hailing from affluent and urban backgrounds are more prone to obesity, recent trends indicate a disproportionate surge in obesity rates among low-income populations, both in rural and urban settings [1]. Various studies have pinpointed socioeconomic correlates of childhood obesity in India, including parental education levels, household income, and the educational institution attended [1,6]. Notably, children enrolled in private schools face a heightened risk of obesity compared to their counterparts in government schools [1]. Moreover, the offspring of working women exhibit a higher susceptibility to obesity than those of non-working women [1]. Additionally, children with a familial history of obesity face an escalated risk of developing the condition [1]. A systematic review and meta-analysis published in 2023 underscored genetic, behavioral, environmental, and socioeconomic determinants contributing to childhood obesity in India [1]. The economic ramifications of childhood obesity in India are profound, as evidenced by a study estimating the annual economic burden of obesity and overweight among Indian children [1]. According to this study, the cost associated with obesity and overweight in Indian children amounted to INR 8,000 crore (approximately USD 1.1 billion) in 2017 [1]. Socioeconomic factors contributing to obesity are shown in Figure 1.

Cultural and Dietary Influences

Cultural and dietary influences exert a profound impact on the prevalence of childhood obesity in India, with various factors at play, including cultural norms, traditional dietary practices, and the influence of globalization. Research studies underscore the significance of cultural influences on child-feeding practices, family dynamics, gender roles, and perceptions of healthy child appearance [7,8]. Moreover, the consumption of energy-dense foods and increased reliance on fast foods, snacks, and sugary beverages, coupled with a shift towards a more sedentary lifestyle, have been identified as key contributors to the escalating childhood obesity rates in India [8,9]. The pervasive availability and affordability of easily accessible yet often unhealthy food options have reshaped dietary habits, exacerbating the prevalence of childhood obesity [10]. The dynamic nature of culture and the rapid pace of cultural change underscore the necessity for culturally sensitive tools and interventions to combat childhood obesity effectively. It is imperative to comprehend and acknowledge the cultural and dietary factors unique to the diverse populations across India to devise comprehensive and sustainable prevention and intervention strategies.

Lack of Physical Activity

Numerous studies have highlighted insufficient physical activity as a prominent contributor to childhood obesity in India [1,9,11]. A comprehensive meta-analysis encompassing 21 studies conducted from 2003 to 2023 involving 186,901 children revealed a pooled prevalence of childhood obesity at 8.4%, albeit with notable heterogeneity. This prevalence exhibits substantial variability across different regions and demographics within India, with rates ranging from 3% to 22.8% among school children aged 6-16 years [1]. Moreover, there has been a concerning upward trend in the occurrence of overweight among children in India, with the prevalence of overweight children under five escalating from 2.1% from 2015 to 2016 to 3.4% in 2019-2021 [4]. The causative factors of childhood obesity in India are attributed to a combination of genetic predispositions, behavioral patterns, environmental influences, and socioeconomic circumstances [1]. In response to this pressing issue, prevention and intervention strategies in India include promoting increased physical activity, curbing sedentary behaviors, implementing personalized nutrition plans, and launching community-based interventions [9,10].

Health implications of childhood obesity

Short-Term Health Effects

Childhood obesity is associated with a multitude of short-term health effects that pose significant risks to affected individuals. These include an elevated likelihood of developing cardiovascular disease, high cholesterol levels, high blood pressure, prediabetes, type 2 diabetes, bone and joint complications, and sleep apnea. Furthermore, children grappling with obesity often contend with social and psychological challenges such as stigmatization and diminished self-esteem [12]. The immediate and long-term repercussions of obesity encompass a broad spectrum of medical ailments, including fatty liver disease, sleep apnea, asthma, cardiovascular complications, elevated cholesterol levels, and orthopedic issues [12,13]. These health conditions not only impact physical well-being but also profoundly affect the psychological health of children and adolescents [12,13]. Addressing childhood obesity is crucial for mitigating these health risks and fostering overall well-being and quality of life.

Long-Term Health Consequences

Childhood obesity carries substantial long-term health ramifications, significantly elevating the risk of numerous serious diseases and health conditions. According to the Centers for Disease Control and Prevention (CDC), individuals grappling with obesity face heightened susceptibility to high blood pressure, high cholesterol, type 2 diabetes, respiratory issues like asthma and sleep apnea, joint ailments, gallbladder disease, and various mental health disorders such as clinical depression and anxiety [14,15]. Additionally, childhood obesity is linked to an augmented likelihood of developing non-communicable diseases later in adulthood. It serves as a risk factor for four of the top 10 leading causes of death in the United States, including coronary heart disease, type 2 diabetes, stroke, and cancer [16]. Moreover, obesity in children and adolescents can engender social and emotional hurdles, including stigmatisation, discrimination, negative stereotypes, diminished academic performance, and a diminished quality of life [14,15]. Therefore, addressing childhood obesity is paramount to alleviate these profound and enduring health implications.

Economic Burden on the Healthcare System

The economic strain posed by healthcare expenses, encompassing medical care costs, insurance premiums, and out-of-pocket spending, carries significant implications for individuals, families, and the healthcare system. A study supported by the National Institutes of Health (NIH) underscored that health disparities within the United States wield a substantial financial toll, with health inequities imposing a considerable burden on the economy [17]. It is noteworthy that the weight of healthcare costs extends beyond the uninsured population, as even individuals with health coverage may encounter difficulties in affording premiums, deductibles, and out-of-pocket expenditures [18]. The economic impact of chronic diseases, including heart disease, stroke, cancer, diabetes, and arthritis, is profound, resulting in elevated medical expenses and diminished productivity [19]. These financial burdens reverberate throughout society, affecting individuals, families, and the broader economy, accentuating the urgency for efficacious strategies to address healthcare affordability and alleviate the economic ramifications of chronic conditions.

Effective prevention strategies

Policy-Level Interventions

Government initiatives and regulations: Policy-level interventions for addressing childhood obesity in India encompass government initiatives and regulations to implement effective strategies. Suggestions include establishing a national task force dedicated to combating obesity, reducing taxes on fruits and vegetables, implementing comprehensive food labelling standards, and ensuring quality monitoring [20]. Furthermore, initiatives may involve the creation of additional playgrounds, parks, and walking/bicycle tracks, coupled with restrictions on the advertisement of commercial foods during prime-time television and children's programs [21]. Encouraging transnational food corporations to produce healthier snack options is also advocated. However, further research is imperative to ascertain the efficacy of these policy-level interventions tailored to the Indian context [5].

School-based programs: School-based intervention programs emerge as pivotal strategies in preventing and managing childhood overweight and obesity in India. These programs typically target diverse facets such as obesity prevention, reduction of excessive consumption of sugary and carbohydrate-rich foods, reduction of sedentary behaviours like excessive television viewing, and promotion of increased physical activity [9]. Moreover, community-based interventions play a crucial role by fostering environments conducive to healthy living, advocating for healthier food alternatives, and enhancing awareness regarding physical activity [22]. It is acknowledged that further research is indispensable to discern the most efficacious strategies for intervention, prevention, and treatment of obesity within the Indian milieu [22].

Community-Level Interventions

Promotion of healthy eating habits: Community-based interventions are pivotal in fostering healthy eating habits and thwarting childhood obesity in India. These initiatives aim to raise awareness and cultivate an environment conducive to adopting a balanced diet and lifestyle. Programs such as "CHETNA" (Centre for Health, Education, Training and Nutrition Awareness) and "MARG" (Medical education for children/Adolescents for Realistic prevention of obesity and diabetes and for healthy aGeing) have been launched, providing nutritional and physical activity education to children, educators, and parents [23]. Additionally, community-level interventions should prioritise promotion of nutritious food options and heighten awareness of the significance of physical activity [23]. These strategies are indispensable for tackling the multifaceted nature of obesity and necessitate collaboration among various stakeholders, including governmental entities, to combat childhood obesity in India effectively [22].

Encouragement of physical activity: Fostering physical activity is a cornerstone in preventing childhood obesity. Physical activity boosts energy expenditure and aids in maintaining a healthy weight [24,25]. Children should have ample opportunities for physical engagement throughout the day, with parents playing a pivotal role in promoting group activities and fostering an environment supportive of physical activity [24]. Schools are instrumental in promoting physical activity by enhancing physical education curricula, providing positive role models for physical engagement, and implementing environment modifications conducive to activity promotion [26]. Moreover, community-based interventions should emphasize the importance of increased physical activity, advocate for healthier food choices, and raise awareness about the benefits of an active lifestyle [26]. Children can augment their physical activity levels through various avenues, including active transportation, unstructured outdoor play, personal fitness endeavors, and organized sports [25,26]. Parents of children in organized sports should be encouraged to facilitate continued physical activity on non-sporting days [26]. Promoting physical activity is a pivotal strategy in preventing childhood obesity in India.

Family-Level Interventions

Education and awareness campaigns for parents: Educational and awareness initiatives targeting parents are pivotal in addressing childhood obesity, equipping them with essential information and resources to instill healthy behaviors in their children. Evidence-based parenting education programs offer structured learning sessions involving parents and caregivers, promoting positive parenting practices, child development, health, nutrition, early learning, and language acquisition [27]. For instance, "La Mallette des Parents" program in France encourages parental involvement in education to enhance pupil outcomes [28]. Similarly, First 5 LA's multiyear Family Strengthening Public Awareness Campaign in the United States furnishes parents and caregivers with essential tips, ideas, and resources to foster optimal child development [29]. Additionally, the "Talk. They Hear You." campaign by Substance Abuse and Mental Health Services Administration (SAMHSA) targets underage drinking and substance use by providing parents with support and resources [30]. Family-based interventions, often incorporating education and awareness components, have proven effective in preventing and managing childhood obesity [31]. These interventions engage families in promoting healthy behaviors among all members, emphasizing behavior change, goal setting, problem-solving, and monitoring children's behaviors [31]. While these initiatives underscore the significance of education and awareness campaigns for parents, further research and culturally tailored approaches are imperative to address childhood obesity effectively, particularly among specific racial and ethnic minority groups [31].

Role of family in fostering healthy habits: A family profoundly influences healthy habits in children, with parents serving as crucial role models whose behaviors significantly shape their children's habits and behaviors. Research underscores that children glean much about eating and physical activity by observing and imitating their parents. Hence, it is paramount for parents to cultivate an environment conducive to healthy choices, such as ensuring ready access to nutritious foods and limiting the availability of unhealthy options at home. Furthermore, positive communication and active involvement in promoting and participating in physical activities with children can significantly influence healthy behaviors. Family-based interventions have effectively promoted and sustained healthy habits in children, constituting a cornerstone strategy for preventing and addressing childhood obesity [32].

Role of Healthcare Professionals

Screening and early intervention: Screening and early intervention are pivotal in addressing childhood obesity, with the US Preventive Services Task Force (USPSTF) recommending screening for obesity in children and adolescents aged six years and older. Those identified with obesity should be offered or referred to family-centred, comprehensive, intensive behavioural interventions to facilitate improvements in weight status [33,34]. The USPSTF has substantiated evidence demonstrating that comprehensive, intensive behavioural interventions (comprising ≥26 contact hours) for children and adolescents aged six years and older with obesity can yield positive outcomes in weight status [33,35]. Effective family-centred care delivery mandates a framework encompassing screening and early detection of obesity and associated risk factors and comorbid conditions [36]. Evidence-based strategies for healthcare providers entail addressing obesity-related concerns, fostering supportive relationships with families, and facilitating access to comprehensive, intensive behavioural interventions to improve weight status [36].

Counseling and support for families: Family counselling, also known as family therapy, serves as a vehicle for cultivating and sustaining healthy and functional family dynamics. It aims to identify and tackle emotional, psychological, or behavioural challenges within the family unit. Family therapy proves effective in addressing a diverse array of issues, spanning communication breakdowns, sibling conflicts, inconsistent parenting practices, marital discord, and adaptation to significant familial changes or crises [37]. The benefits of family counselling encompass establishing healthy boundaries, enhancing communication skills, clarifying familial roles, ameliorating dynamics and relationships, and providing coping mechanisms for family members [37]. Typically conducted in collaboration with a therapist, family therapy can significantly fortify relationships and is particularly beneficial during tumultuous periods or transitions [38]. It may be integrated with other therapeutic modalities such as individual child or adolescent therapy, group counselling, or couples therapy, incorporating interactive activities and play-based strategies to engage all family members [38]. Moreover, family counselling can be administered through diverse formats, including support groups, one-on-one sessions with a therapist, or structured family counselling to bolster healthy behaviors, nurture emotional growth, and foster improvements in familial and peer relationships [39,40].

Challenges and barriers

Socioeconomic Disparities

Socioeconomic disparities significantly contribute to childhood obesity in India, with children from low-income and low-education households being more susceptible to obesity [41,42]. Moreover, Hispanic, African American, and American Indian children face more significant disadvantages compared to their Asian and White counterparts [42]. The coexistence of undernutrition and obesity in India presents a unique challenge, with different socioeconomic strata encountering diverse obstacles [43]. Economic barriers, including time and financial constraints, pose significant challenges for parents in adopting obesity prevention recommendations [41]. Families with limited resources are more likely to reside in communities characterised as "food deserts," where access to grocery stores is limited while fast-food restaurants and convenience stores proliferate [44]. To address socioeconomic disparities in childhood obesity, interventions must contextualise the country's economic, social, and cultural milieu, devising comprehensive, multidisciplinary, and evidence-based strategies that acknowledge diverse barriers [41,42]. Actionable approaches include promoting exclusive breastfeeding and home-cooked meals for children, implementing restrictions on fast food within households and schools, and ensuring regular breakfast consumption [43].

Cultural Resistance to Change

Cultural resistance to change poses a significant barrier in addressing childhood obesity, as cultural beliefs and practices can profoundly influence perceptions and decisions, potentially exacerbating the issue [45]. Body image development, for instance, occurs within a cultural framework, and diverse ethnic or cultural groups may harbour varying ideals regarding body size, consequently shaping attitudes towards obesity [8]. Furthermore, cultural values, beliefs, and dietary customs can foster lifestyles conducive to obesity among children and adolescents [45]. Recognising these cultural nuances is imperative when devising interventions, as culturally tailored strategies are indispensable for enhancing health promotion endeavours and effectively tackling childhood obesity [45]. Hence, interventions must be meticulously tailored to the cultural context to surmount resistance to change and effectively address childhood obesity.

Lack of Infrastructure and Resources

The lack of infrastructure and resources poses a significant hurdle in combating childhood obesity in India, compounded by the coexistence of undernutrition and obesity, presenting a multifaceted challenge across different socioeconomic strata [44]. Families with limited means often reside in "food deserts," where nutritious options are scarce while fast-food outlets proliferate [44]. Moreover, challenges extend to working parents with demanding schedules or financial constraints, hindering their ability to adopt obesity prevention measures [44]. A dearth of awareness, coupled with misconceptions and the absence of targeted policies and programs addressing childhood obesity, exacerbates the issue [46]. India's ranking of 99th out of 183 countries regarding preparedness to tackle obesity underscores the pressing need for action [46]. Addressing these challenges mandates interventions that consider the nation's economic, social, and cultural fabric, necessitating comprehensive, multidisciplinary, evidence-based strategies [46,47]. Effecting change would require concerted efforts from government, industry, and civil society stakeholders [43].

Marketing and Availability of Unhealthy Foods

**Figure 2:**
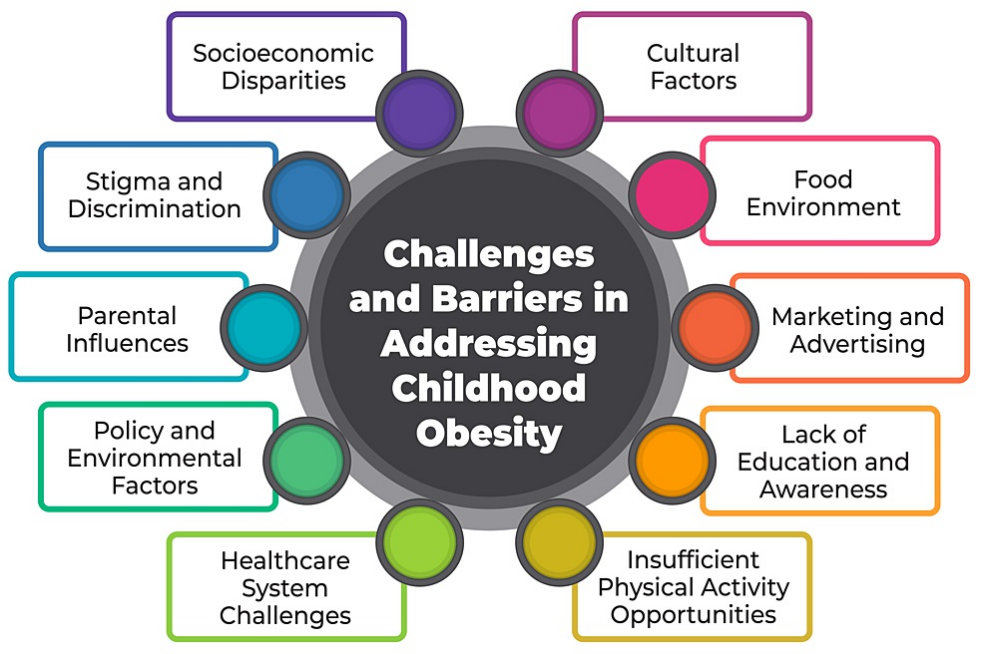
Challenges and barriers

The marketing and widespread availability of unhealthy foods pose a significant challenge when addressing childhood obesity in India, where the burgeoning market for junk food lacks stringent marketing regulations, particularly concerning children [48]. Starchy cereals and sugary beverages are promoted as premium options through advertisements featuring Bollywood celebrities and cartoon characters, contributing to a nearly tripled sales volume of snacks and soft drinks over the past decade [48]. This landscape is further compounded by a lack of awareness, misperceptions, and the absence of targeted policies and programs directly addressing childhood obesity [49]. An analysis of junk food advertisements in India highlighted a glaring absence of crucial nutritional information regarding sugar, salt, and fat content in these products. To confront this challenge, there have been fervent calls for banning junk food advertising in the country [49]. Implementing strategies such as policy formulation, establishing a national task force for obesity, reducing taxes and prices on fruits and vegetables, ensuring proper food labelling practices, quality monitoring, and bolstering recreational infrastructure with more playgrounds, parks, and walking and bicycle tracks are paramount [48]. Encouraging transnational food corporations to diversify into healthier snack options is also advocated to counter the prevalence of unhealthy food choices [48]. Challenges and barriers in addressing childhood obesity are shown in Figure 2.

## Conclusions

In conclusion, this review underscores the critical importance of addressing childhood obesity in India through comprehensive and multifaceted strategies. Key findings highlight the necessity of collaborative efforts among stakeholders and policymakers to implement evidence-based interventions tailored to the Indian context. These interventions should encompass policy-level initiatives, community-based programs, and family involvement to effectively combat the rising prevalence of childhood obesity. Given the significant health, economic, and social implications associated with childhood obesity, prioritizing prevention efforts is paramount. It is imperative for stakeholders and policymakers to recognize the urgency of this issue and take decisive action to promote healthy lifestyles, create supportive environments, and allocate resources for prevention programs. By working together and implementing targeted interventions, India can make significant progress in reducing childhood obesity rates and improving the overall health outcomes of its population.
